# Genome-wide association study of body weight in Australian Merino sheep reveals an orthologous region on OAR6 to human and bovine genomic regions affecting height and weight

**DOI:** 10.1186/s12711-015-0142-4

**Published:** 2015-08-14

**Authors:** Hawlader A. Al-Mamun, Paul Kwan, Samuel A. Clark, Mohammad H. Ferdosi, Ross Tellam, Cedric Gondro

**Affiliations:** School of Environmental and Rural Science, University of New England, Armidale, NSW 2351 Australia; School of Science and Technology, University of New England, Armidale, NSW 2351 Australia; CSIRO Animal, Food and Health Sciences, Queensland Bioscience Precinct, St. Lucia, QLD 4067 Australia

## Abstract

**Background:**

Body weight (BW) is an important trait for meat production in sheep. Although over the past few years, numerous quantitative trait loci (QTL) have been detected for production traits in cattle, few QTL studies have been reported for sheep, with even fewer on meat production traits. Our objective was to perform a genome-wide association study (GWAS) with the medium-density Illumina Ovine SNP50 BeadChip to identify genomic regions and corresponding haplotypes associated with BW in Australian Merino sheep.

**Methods:**

A total of 1781 Australian Merino sheep were genotyped using the medium-density Illumina Ovine SNP50 BeadChip. Among the 53 862 single nucleotide polymorphisms (SNPs) on this array, 48 640 were used to perform a GWAS using a linear mixed model approach. Genotypes were phased with *hsphase*; to estimate SNP haplotype effects, linkage disequilibrium blocks were identified in the detected QTL region.

**Results:**

Thirty-nine SNPs were associated with BW at a Bonferroni-corrected genome-wide significance threshold of 1 %. One region on sheep (*Ovis aries*) chromosome 6 (OAR6) between 36.15 and 38.56 Mb, included 13 significant SNPs that were associated with BW; the most significant SNP was OAR6_41936490.1 (*P* = 2.37 × 10^−16^) at 37.69 Mb with an allele substitution effect of 2.12 kg, which corresponds to 0.248 phenotypic standard deviations for BW. The region that surrounds this association signal on OAR6 contains three genes: *leucine aminopeptidase 3* (*LAP3*), which is involved in the processing of the oxytocin precursor; *NCAPG non-SMC condensin I complex, subunit G* (*NCAPG)*, which is associated with foetal growth and carcass size in cattle; and *ligand dependent nuclear receptor corepressor-like* (*LCORL*), which is associated with height in humans and cattle.

**Conclusions:**

The GWAS analysis detected 39 SNPs associated with BW in sheep and a major QTL region was identified on OAR6. In several other mammalian species, regions that are syntenic with this region have been found to be associated with body size traits, which may reflect that the underlying biological mechanisms share a common ancestry. These findings should facilitate the discovery of causative variants for BW and contribute to marker-assisted selection.

**Electronic supplementary material:**

The online version of this article (doi:10.1186/s12711-015-0142-4) contains supplementary material, which is available to authorized users.

## Background

In sheep, body weight (BW) is an important economic trait for meat production. Currently, genome-wide association studies (GWAS) are applied to identify candidate genes for many quantitative traits, not only in sheep, but also in many other species [1–6]. Several GWAS for growth and meat production traits in cattle have been published in recent years [7–9]. GWAS for bovine carcass weight and other production traits have revealed major quantitative trait loci (QTL) on chromosomes (BTA for *Bos taurus*) BTA6, 8, 11, 14, 24 and 25 [10–12]. For carcass weight, QTL have been reported in a 1.1 Mb region on BTA14 and a 591 kb region on BTA6 by Mizoshita et al. [10] and Setoguchi et al. [12], respectively. Nishimura et al. [11] also identified three major QTL for carcass weight in Japanese black cattle on BTA6, 8 and 14, which together explained approximately one-third of the genetic variance of carcass weight. Karim et al. [13] mapped a QTL to a 780 kb region on BTA14 that had a major effect on bovine stature. The QTL region on BTA14 contains six genes that are associated with stature in both cattle and humans [14–17]: *pleiomorphic adenoma gene 1* (*PLAG1*), *coiled-coil-helix-coiled-coil-helix domain containing 7* (*CHCHD7*), *proenkephalin* (*PENK*), *V-Mos Moloney murine sarcoma viral oncogene homolog* (*MOS*), *V-Yes-1 Yamaguchi sarcoma viral related oncogene homolog* (*LYN*) and *trimethylguanosine synthase 1* (*TGS1*). The same QTL region on BTA14 has also been shown to affect birth weight and size in zebu cattle (*Bos primigenius indicus*) [18]. In addition, Lee et al. [19] detected a major QTL on BTA14 for carcass weight in Korean cattle (Hanwoo), which explained at least 10 % of the genetic variation of carcass weight in this breed. In brown Swiss cattle, 74 genome-wide significant single nucleotide polymorphisms (SNPs) were shown to be associated with one or more production traits, including fertility, conformation, udder health and workability on BTA6, 11, 24 and 25 [20].

Although many GWAS have identified important candidate genes in different species, there are comparatively few QTL studies for sheep. To date, 801 ovine QTL are curated in the Sheep QTLdb [21], among which only a very small number concern meat production. Most of the QTL studies that have been performed on sheep were based on sparse microsatellite markers that exhibited large QTL confidence intervals, which makes it very difficult to identify candidate genes for the targeted quantitative trait. In sheep, few GWAS have focused on growth and meat production traits [22–24] and among these, only one study reported a GWAS and fine mapping of QTL for BW, on ovine (*Ovies aries*) chromosome 21 (OAR21) [24].

In this study, our aims were (1) to perform a GWAS to detect significant SNPs that are associated with BW in sheep by using data from 1781 Australian Merino sheep genotyped with the Illumina Ovine SNP50 BeadChip and (2) to explore the genomic regions around these SNPs for candidate genes.

## Methods

### Ethical statement

Samples for genotyping were collected under approval number 344 AEC12-049 of the University of New England Animal Ethics Committee.

### Phenotypic data

The data used in this study consisted of phenotypic records from 1781 half-sib family groups of Merino sheep from the Australian Sheep CRC Information Nucleus flock, which included 1088 males and 693 females sired by 111 sires. The maximum and minimum numbers of sheep per sire group were 49 and 1, respectively. Post-weaning weight, which is an early life body weight measurement, was measured at an average age of 287.5 days, with a minimum of 148 days and a maximum of 431 days. Body weights ranged from 17.5 to 67.2 kg, with an average of 39.7 kg, and were normally distributed.

### Genotyping and quality control

All animals were genotyped using the Illumina Ovine SNP50 BeadChip (Illumina Inc., San Diego, CA, USA), which includes 53 862 SNPs. Quality control was performed with the R program *snpQC* and SNPs were filtered as follows: SNPs were removed if they had a call rate less than 95 %, a GenCall score less than 0.6, a minor allele frequency lower than 0.01 and if their heterozygosity rate was outside the interval of the mean ± 3 × standard deviations (mean heterozygosity rate = 0.37 and s.d. = 0.13). SNPs that departed from Hardy-Weinberg equilibrium (for a *P*-value cut-off of 1 × 10^−15^) or had no assigned genomic location or were located on the sex chromosomes were also excluded from the analyses. Missing genotypes were imputed using fast PHASE [25].

### Statistical analyses

A GWAS was performed with a linear mixed effects model using ASREML [26]. The following fixed effects were fitted in the model: sex, birth type, rearing type, age of dam, contemporary group (birth year * birth month * site * management group) and age at trait recording. To account for family effects, sire was also fitted as a random effect (sire model with pedigree). The additive allelic substitution effect was individually calculated for each SNP by fitting the following mixed model:

1 $$ \mathbf{y}=\mathbf{X}\mathbf{b}+\mathbf{Zg} + \upvarepsilon, $$

where, **y** is a vector of individual body weights, **X** is a design matrix for fixed effects (as described above) and SNP genotypes fitted as covariate, and **b** is a vector of fixed and SNP effects. **Z** is a matrix that allocates records to sire, **g** is a vector of sire effects, and ε is the residual. After fitting the data in model (1), analysis of the residuals indicated that the model was reliable and did not violate the statistical assumption of normality. *P*-values were adjusted for multiple-testing to a 0.01 Bonferroni-corrected significance threshold (*P* < 2.05 × 10^−7^). To evaluate if estimates were overinflated, we calculated the genomic inflation factor λ using the GenABEL R package [27].

The percentage of genetic variance explained by each significant SNP was calculated according to the following formula:

2 $$ \%{V}_{gi}=100\times 2{p}_i{q}_i{a_i}^2/{\sigma_g}^2, $$

where *p*_*i*_ and *q*_*i*_ are the allele frequencies for the *i*^*th*^ SNP, *a*_*i*_ is the estimated additive effect of the *i*^*th*^ SNP on BW, and *σ*_*g*_^*2*^ is the estimated genetic variance. ASReml [26] was used to estimate heritability (*h*^2^) and genetic variance for BW using the same data and model as Eq. 1, however without including SNP effects.

Estimation of the genetic variance attributable to each individual chromosome was done using the GCTA [28] software. A genomic relationship matrix was built for each of the 26 autosomes using only the SNPs mapped to each of the chromosomes. All chromosomes were then fitted simultaneously in GCTA using the 26 GRM.

### Identification of haplotype blocks and regression analyses

The *hsphase* algorithm [29] was used to reconstruct haplotypes on OAR6. Since this algorithm relies on a minimum number of individuals in a half-sib family, we opted for a conservative approach and excluded half-sib families that had less than 10 individuals. Thus, paternal and maternal haplotypes for OAR6 were phased for 1569 individuals. Missing alleles in the haplotypes were imputed with BEAGLE 3.3.2 [30]. Finally, the regions that contained significant SNPs were selected for haplotype analyses (45 SNPs in the region between 36.15 and 38.56 Mb).

Linkage disequilibrium (LD) between SNPs within the region between 36.15 and 38.56 Mb on OAR6 was calculated as |D’|, using Haploview (V4.2 [31]). LD blocks were generated for SNPs that were separated by less than 500 kb, as proposed by Gabriel et al. [32]. Haplotype association analyses were performed using the following multiple regression mixed model equation:

3 $$ {y}_j=\mu +{\displaystyle \sum_{i=1}^t}{\beta}_{ij}{H}_i+{S}_j+{e}_{ij}, $$

where *y*_*j*_ is the residual phenotypic value for the *j*^*th*^ individual after adjusting for the fixed effects (Eq. 1); *μ* is the overall mean; *β*_*ij*_ is the haplotype score (0, 1, or 2) of the *i*^*th*^ haplotype for the *j*^*th*^ individual, with *t* the number of haplotypes segregating in the population for that region; *H*_*i*_ is the effect of the *i*^*th*^ haplotype; *S*_*j*_ is the random sire effect, and *e*_*ij*_ is the random residual effect.

## Results

### Descriptive statistics and quality control

From the initial set of 53 862 SNPs, 1449 (2.69 %) non-autosomal SNPs, 1662 SNPs with a minor allele frequency lower than 0.01, 1838 SNPs with a call rate (CR_SNP_) less than 0.95, and 273 SNPs that departed from the Hardy-Weinberg equilibrium were removed. No individual was excluded because of a call rate (CR_IND_) lower than 0.7, but 38 individuals were excluded because of missing phenotypes. After quality control, 1743 individuals and 48 640 SNPs were retained for analysis.

The number and the average distances between adjacent SNPs on each chromosome, before and after filtering, are in Table S1 [See Additional file 1: Table S1]. Before quality control, the number of SNPs on each chromosome ranged from 741 on OAR24 to 5930 on OAR1, while average adjacent distances ranged from 44.1 kb on OAR8 to 56.8 kb on OAR24. After quality control, the number of SNPs on each chromosome ranged from 679 on OAR24 to 5494 on OAR1 and average adjacent distances ranged from 47.2 kb on OAR8 to 66.2 kb on OAR21.

### Association analyses

**Fig. 1:**
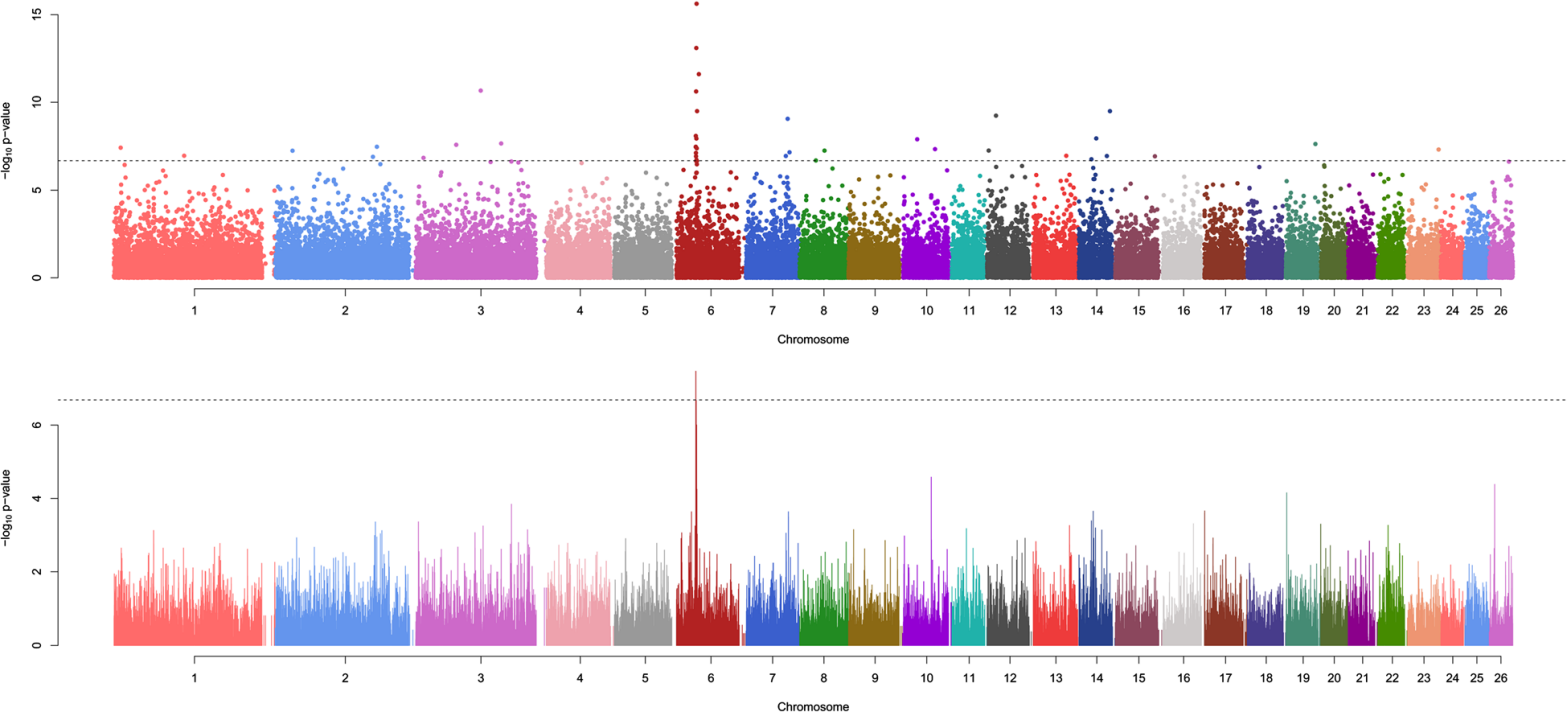
Manhattan plots. Manhattan plots of genome-wide -log_10_ (*p*-values) for body wei*ght in Australian Merino sheep. Top panel – individual SNP results; lower panel – smoothed median P*-values using five SNP intervals

**Fig. 2:**
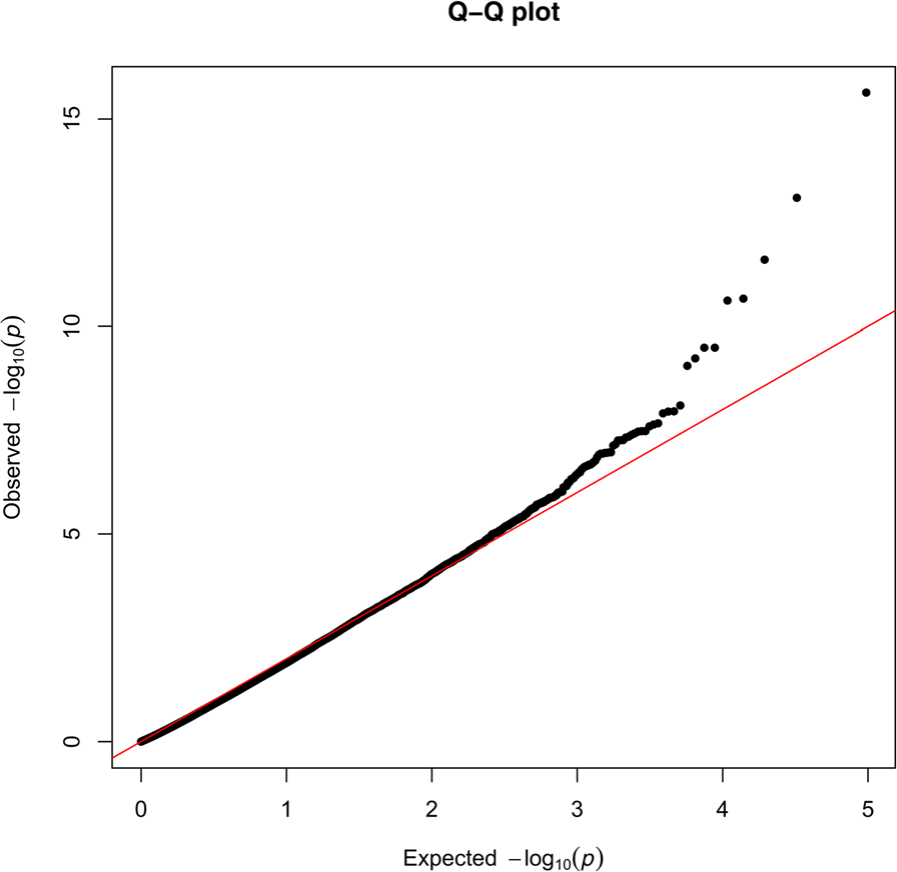
Quantile-quantile plot. Quantile-quantile plot for -log_10_ (*P*-value) for the association analysis (λ = 1.13). Black dots represent the -log_10_(*P*-value) of the entire study and the red line represents the expected values under the null hypothesis of no association

The GWAS identified one major region with a strong association with BW between 36.15 and 38.56 Mb on OAR6 (Fig. 1 top panel). To reduce the detection of spurious associations, GWAS results were smoothed with a running median of the *P*-values that spanned five adjacent SNPs (Fig. 1 lower panel). For SNPs with *P*-values less than 0.01, the quantile-quantile (Q-Q) plot revealed a large deviation from the distribution under the null hypothesis, which indicated a strong association between this region and BW (Fig. 2). Significant Bonferroni-corrected genome-wide associations (*P* < 2.05 × 10^−7^) were detected for 39 SNPs [See Additional file 2: Table S2], of which 13 were located on OAR6 (Table 1). Among the five most significant SNPs, four were located on OAR6 and, out of these, the most significant was OAR6_41936490.1 (*P* = 2.36 × 10^−16^), which is located at 37.69 Mb. The other three SNPs on this chromosome were s17946.1 (*P* = 7.97 × 10^−14^) at 37.1 Mb, OAR6_41877997.1 (*P* = 2.49 × 10^−12^) at 37.64 Mb, and OAR6_41003295.1 (*P* = 2.4 × 10^−11^) at 36.81 Mb. Apart from this region on OAR6, the most significant SNP was on OAR3, i.e. OAR3_128968872.1 (*P* = 2.16 × 11^−16^) at 120.9 Mb. Four SNPs were detected on OAR14, at 23.5, 32.4, 52.2 and 57.9 Mb. The remaining significant SNPs were spread over various chromosomes [See Additional file 2: Table S2]. After smoothing, only the region between 36.15 and 38.56 Mb on OAR6 exceeded the significance threshold. In this region, there were 45 SNPs on the array, of which 30 were significantly associated with BW prior to Bonferroni correction (unadjusted *p*-value < 0.001).

**Table 1:** SNPs on OAR6 showing significant association with body weight in 1743 Merino sheep

SNP	Position	Adjusted *P*-value	n0	n1	n2	p	q	v_g_	β (kg)
OAR6_41936490.1	37694563	1.15 × 10^−11^	119	706	918	0.271	0.729	6.17	2.116
s17946.1	37164383	3.88 × 10^−09^	83	630	1030	0.228	0.772	5.88	−2.132
OAR6_41877997.1	37640732	1.21 × 10^−07^	110	650	983	0.250	0.750	6.05	−1.876
OAR6_41003295.1	36819342	1.17 × 10^−06^	150	722	871	0.293	0.707	6.50	−1.741
OAR6_42945420.1	38567455	1.56 × 10^−05^	22	321	1400	0.105	0.895	6.54	2.28
OAR6_40449774.1	36234302	3.87 × 10^−04^	20	378	1345	0.120	0.880	6.62	−2.079
OAR6_41558126.1	37334387	5.45 × 10^−04^	79	574	1090	0.210	0.790	6.30	1.686
OAR6_40409402.1	36192023	1.61 × 10^−03^	9	254	1480	0.078	0.922	6.65	2.337
OAR6_40370293.1	36155169	1.65 × 10^−03^	110	641	992	0.247	0.753	6.25	−1.589
OAR6_42247197.1	37987281	1.97 × 10^−03^	60	512	1171	0.181	0.819	6.52	−1.645
OAR6_40724811_X.1	36522166	3.61 × 10^−03^	18	385	1340	0.121	0.879	6.26	−1.914
OAR6_40855809.1	36655091	5.65 × 10^−03^	53	448	1242	0.159	0.841	6.34	1.612
OAR6_41768532.1	37533664	9.01 × 10^−03^	64	521	1158	0.186	0.814	6.46	1.542

Thirteen SNPs on OAR6 reach the genome-wide significance level (adjusted *P*-value < 0.01). These SNPs span the region between 36.15 and 38.56 Mb. A total of 45 SNPs on the Illumina Ovine SNP50 BeadChip are mapped to this region and 30 were identified as significant for an unadjusted *p*-value threshold of 0.001. n0, n1, and n2 are the number of individuals with 2, 1 and 0 copies of the minor allele; p is the minor allele frequency and q = 1 – p; Vg indicates the proportion of the genetic variance that is attributable to each SNP estimated by ASReml (note that SNPs are not unlinked and the sum of effects will be overestimated). β is the allele substitution effect. SNP positions are based on the Oar_v3.1 assembly of the ovine genome sequence

**Fig. 3:**
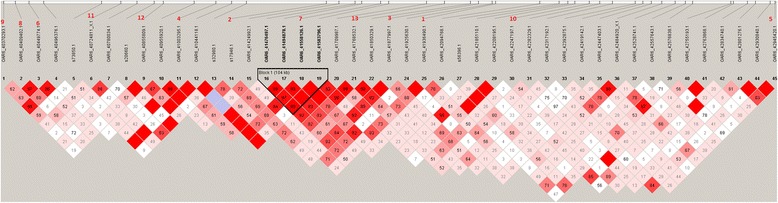
Linkage disequilibrium (LD) map. Extent of LD across the 2.41 Mb region between 36.15 and 38.56 Mb on OAR6. At the top of the figure, the 13 significant SNPs identified in this region are highlighted in red by order of significance. The haplotype block of 104 kb containing four SNPs is shown in bold black

The patterns of LD in the region between 36.15 and 38.56 Mb on OAR6 were investigated using |D’| (Fig. 3). One clear LD block of 104 kb was detected, with LD blocks defined following the criteria described in [32]). This block contained one of the genome-wide significant SNPs (7^th^ ranked), while the second most significant SNP was on its border (Fig. 3).

**Fig. 4:**
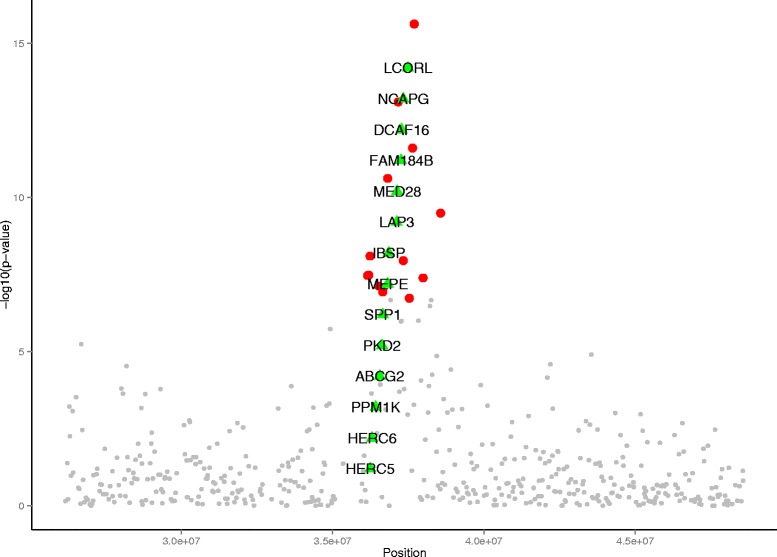
Plot of candidate genes in the QTL region. Plot of the *p*-values in a 20 Mb region on OAR6 and surrounding candidate genes. Significance (-log_10_ of *P*-values) is plotted against positions along the OAR6. A total of 13 significant SNPs are indicated by larger red dots and the candidate genes present in this region are indicated by green triangles. Remaining SNPs are indicated by smaller grey dots

**Fig. 5:**
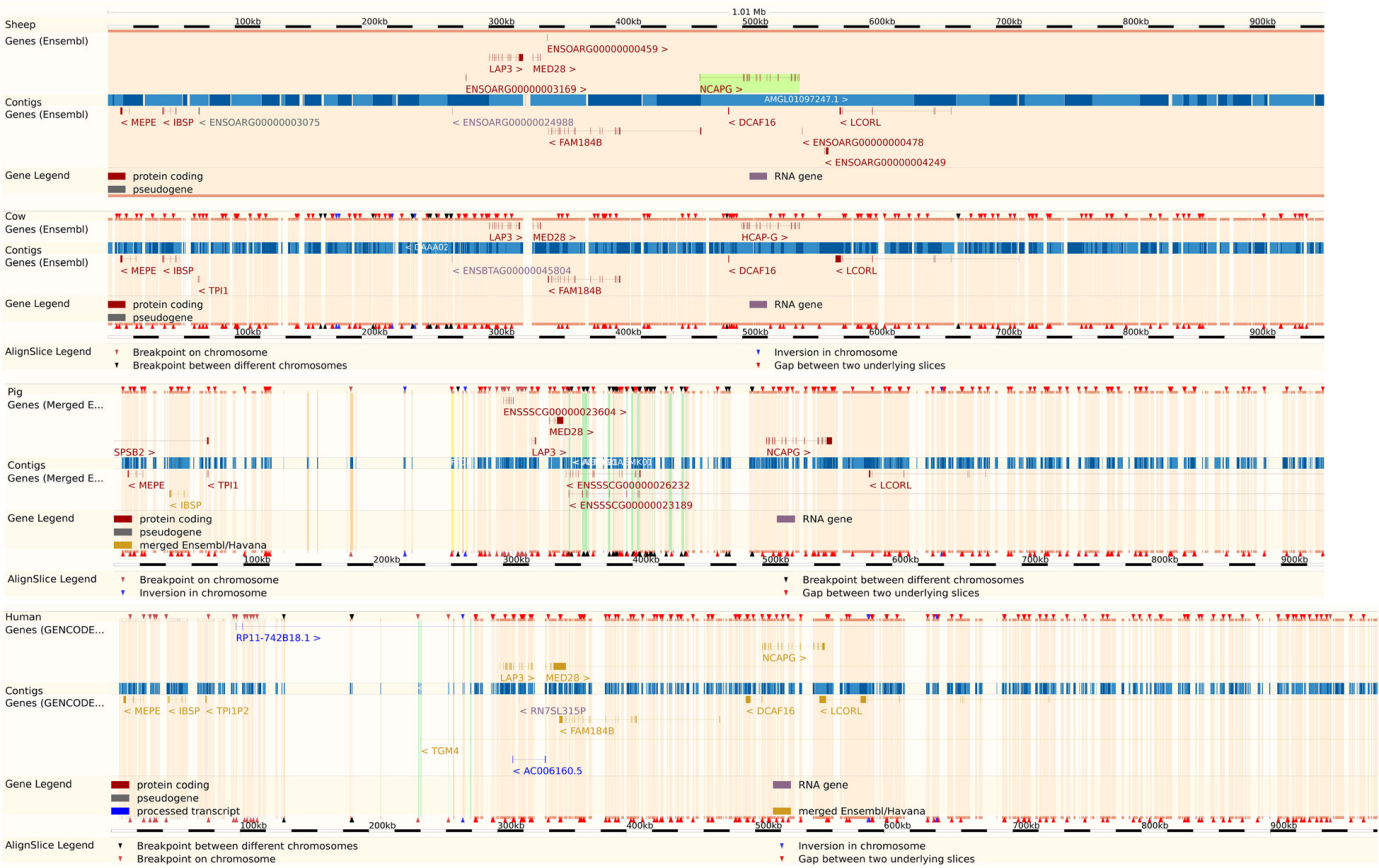
Ensembl alignments of the sequence for the 1 Mb region surrounding the genes *NCAPG and LCORL*. The *Ovis aries* reference genome (Oar_v3.1 assembly) was aligned against (from top to bottom) bovine (UMD v3.1 assembly), porcine (Sscrofa 10.2 assembly) and human (GRCh37 assembly) genomes. Code colour for genes: brown – protein coding, grey – pseudo gene, blue – processed transcript, yellow – merged Ensembl/Havana and purple – RNA gene. Triangles: black – breakpoint between different chromosomes, blue – inversion in chromosome, brown – breakpoint on chromosome and red – gap between two underlying slices

Based on the *Ovis aries* reference genome assembly (Oar_v3.1), the region between 36.15 and 38.56 Mb on OAR6 contains 14 genes. These include *NCAPG non-SMC condensin I complex, subunit G* (*NCAPG)*, which is associated with foetal growth and carcass size in cattle [33], and *ligand dependent nuclear receptor corepressor-like* (*LCORL*), which is associated with height in humans and cattle [14, 15] (Table 2 and Fig. 4). The *contig* tracks in Fig. 5 show that a high level of sequence conservation was found between the 1.0 Mb sequence of the OAR6 region around *NCAPG and LCORL* and the syntenic regions on BTA6, HSA4 (HSA for *Homo sapiens* chromosome) and SSC8 (SSC for *Sus scrofa* chromosome). When considering a larger 2.41 Mb region (Table 2), more genes were identified, as well as 12 previously reported QTL using different sheep breeds [21] (Table 3). These included QTL for average daily gain, body weight, milk yield, milk lactose yield, and milk fat percentage.

**Table 2:** List of known genes in the 36.15–38.56 Mb region on OAR6

Gene symbol	NCBI Gene ID	OAR6 coordinates (bp)	Distance from most significant SNP (kb)	HSA4 homology	BTA6 homology	SSC8 homology	Gene description
*HERC5*	101119562	36197360..36244346	1450	51191	514373	100518083	HECT and RLD domain containing E3 ubiquitin protein ligase 5
*HERC6*	101103321	36252541..36306667	1387	55008	527520	100626657	HECT and RLD domain containing E3 ubiquitin protein ligase family member 6
*PPM1K*	101119821	36397745..36421249	1273	152926	540329	100723717	protein phosphatase, Mg^2+^/Mn^2+^ dependent, 1K
*ABCG2*	780508	36445816..36556892	1137	9429	536203	397073	ATP-binding cassette, sub-family G (WHITE), member 2
*PKD2*	101103569	36564403..36630798	1063	5311	530393	641309	polycystic kidney disease 2 (autosomal dominant)
*SPP1*	443058	36645289..36658205	1036	6696	281499	397087	secreted phosphoprotein 1
*MEPE*	101120244	36804572..36819432	875	56955	613958	100521206	matrix extracellular phosphoglycoprotein
*IBSP*	101120495	36837345..36850895	843	3381	281233	397137	integrin-binding sialoprotein
*LAP3*	101120750	37092323..37116174	578	51056	781648	100739583	leucine aminopeptidase 3
*MED28*	101121011	37126382..37132492	562	80306	513972	425350	mediator complex subunit 28
*FAM184B*	101121264	37138515..37257047	437	27146	523874	100627969	family with sequence similarity 184, member B
*DCAF16*	101121517	37278542..37279451	415	54876	777600	100513483	DDB1 and CUL4 associated factor 16
*NCAPG*	101121775	37289107..37333949	360	64151	531234	100513670	non-SMC condensin I complex, subunit G
*LCORL*	101104320	37362103..37488824	205	254251	540095	100337668	ligand dependent nuclear receptor corepressor-like

**Table 3:** QTLdb hits within regions surrounding the 36.15-38.56 Mb interval on OAR6

Trait	OAR6 Coordinate	QTLdb ID	Pubmed ID
Average daily gain (between 56 and 83 weeks)	33609760:33760089	13957	19389264 [51]
Body weight (at 83 weeks)	42319448:42469778	13934	19389264
Average daily gain (between 43 and 56 weeks)	42319448:42469778	13950	19389264
Body weight (at 43 weeks)	39786167:39936497	13923	19389264
Milk yield	43302377:51178327	13818	19320771 [52]
Milk lactose yield	43302377:51178327	13819	19320771
Milk yield	43302377:51178327	13820	19320771
Milk lactose yield	43302377:51178327	13821	19320771
Milk fat percentage	6458777:36327544	14243	21749424 [53]
Milk fat percentage	6458777:36327544	14244	21749424
Milk fat percentage	6458777:36327544	14245	21749424
Milk yield	6458777:36327544	14246	21749424

In our analysis, we calculated the proportion of additive genetic variance explained by each SNP for a Bonferroni adjusted *P*-value cut off threshold of 0.01, detected by single-marker regression analysis. Although this variance could be overestimated because polygenic effects may not be fully accounted for, the most significant SNP explained a relatively large percentage (7.22 %) of the total additive genetic variance. The estimated heritability (*h*^2^) for BW was equal to 0.63.

**Fig. 6:**
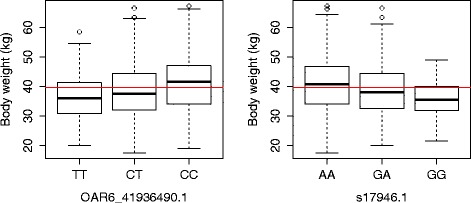
Estimates of SNP effects. Boxplots of the genotypes of the two SNPs that are most significantly associated (OAR6_41936490.1 and s17946.1) with body weight in the Australian Merino sheep. The horizontal red line shows overall population mean weight values and lines within the boxes are the median weights within a genotypic group. There were few extreme values (outliers), which are indicated by circles. Note that both SNPs are in LD and, most likely, they track the same QTL

Allele substitution effects for the 13 significant SNPs on OAR6 ranged from 1.54 to 2.34 kg. The most significant SNP (*OAR6_41936490.1*; *P* = 2.36 × 10^−16^) had an allele substitution effect of 2.12 kg, while the second most significant SNP (*s17946.1*; *P* = 7.97 × 10^−14^) had an effect of 2.13 kg, corresponding respectively to 24.82 and 29.5 % of the phenotypic standard deviation for BW (Table 1 and Fig. 6). However, it should be noted that these two SNPs are in LD and most likely track the same QTL.

We also calculated the percentage of genetic variance explained by each chromosome and found that OAR6 explained 7.71 % of the additive genetic variance [See Additional file 3: Table S3]. Although OAR12 explained the largest proportion of the variance, i.e. 8.91 %, only two significant SNPs were detected on this chromosome by single-SNP regression analysis (Fig. 1).

### Haplotype analyses

A haplotype analysis of the region between 36.15 and 38.56 Mb on OAR6 was carried out and SNPs that were in high LD within this region were grouped together in haplotype blocks. Using the criteria specified by Gabriel et al. [32], a haplotype block of 104 kb was identified. This haplotype block contains four SNPs (OAR6_41476497.1, OAR6_41494878.1, OAR6_41558126.1, OAR6_41583796.1), which were all significantly associated with BW prior to Bonferroni correction (unadjusted *p*-value ≤ 0.0001). |D’| values between SNPs in this block ranged from 0.97 to 1.00 (Fig. 3). Seven distinct haplotypes were detected in this block. Using Eq. (3), these haplotypes were shown to have a highly significant (*p*-value < 0.0008) effect on sheep BW. Haplotypes AGGT and GATC showed significant but opposite effects, while haplotype GATT had a marginal effect on BW (Table 4). By visual inspection (Fig. 3), we found that two other neighbouring SNPs, outside the haplotype block, were also in high LD with the SNPs in this haplotype block. Thus, we extended the haplotype blocks to include these two additional SNPs in the haplotype analysis. We found that these haplotype blocks also had significant associations with BW, with a *p*-value less than 0.001 for a block of 223 kb (SNPs OAR6_41476497.1, OAR6_41494878.1, OAR6_41558126.1, OAR6_41583796.1 and OAR6_41709987.1), and less than 0.006 for a block of 278 kb (SNPs OAR6_41476497.1, OAR 6_41494878.1, OAR6_41558126.1, OAR6_41583796.1, OAR6_41709987.1 and OAR6_41768532.1).

**Table 4:** Estimates of haplotype effects of the QTL for body weight

Haplotype	β^a^	*P*-value^b^
AATC	0.166	0.86752
AGTT	−0.095	0.27794
AGGT	0.049	0.00155
GATT	−0.164	0.01857
GATC	−0.158	0.00288
GAGT	−0.330	0.23310
GGGT	0.451	0.19978

The *P*-value of the haplotype effect in a linear mixed model including a mean, a random sire effect and multiple regressions on the haplotypes was equal to 0.0008

^a^Estimates of regression coefficients (β) in phenotypic standard deviation (STD) units of the trait based on phenotypes

^b^ *P*-values for the test of β ≠ 0

## Discussion

In this study, we performed a GWAS on Australian Merino sheep by genotyping data using a medium-density chip that included around 50 000 SNPs. The analysis identified 13 SNPs that spanned a 2.41 Mb region on OAR6 that were significantly associated (P < 0.001) with BW. Biologically relevant genes in this region are *ATP-binding cassette sub-family G member 2* (*ABCG2*), *polycystin-2* (*PKD2*), *leucine aminopeptidase 3* (*LAP3*), *NCAPG non-SMC condensin I complex, subunit G* (*NCAPG*), and *ligand dependent nuclear receptor corepressor-like* (*LCORL*). Chromosomal locations and known functions of these genes are in Table 2. A causative mutation in the *ABCG2* gene was previously reported to affect milk yield and composition in dairy cattle [34]. The *LAP3* gene encodes leucine aminopeptidase, which is associated with milk production traits in cattle [35]. In humans, the *LCORL* gene encodes a ligand dependent nuclear corepressor-like transcription factor and polymorphisms in this gene are associated with skeletal frame size and adult height (http://www.ncbi.nlm.nih.gov/). In cattle, a non-synonymous but chemically conserved variant in the *NCAPG* gene has been identified as a potential causative variant for body frame size [36]: height, length and width at puberty. However, this variant was excluded as the causal mutation for the QTL that affects fetal growth and carcass traits, and is located in the same region using data on a Charolais x Holstein cross [37]. Similarly, SNPs located in the equine *NCAPG* and *LCORL* genes have been reported to be associated with several body size traits [5].

We used LD to identify haplotype blocks associated with BW. In the region between 36.15 and 38.56 Mb on OAR6, a haplotype block of four SNPs was identified that had a significant effect on BW in sheep. This haplotype block ranged from 37 254 883 to 37 359 421 bp and contained two functional candidate genes: *NCAPG* between 37 362 103 and 37 333 949 bp and *LCORL* between 37 362 163 and 37 488 824 bp. Results are in agreement with several previous studies on different species, including sheep, cattle, horse and chicken. In a study on Scottish Blackface lambs, Riggio et al. [22] reported a region between 33.2 and 37.7 Mb on OAR6 that contained several SNPs associated with BW at different ages (from 6 to 24 weeks old). This result agrees with the 36.15–38.56 Mb region on OAR6 that we found to be significantly associated with BW. In Australian sheep, Daetwyler et al. [38] reported that SNP OAR6_41936490.1 (the most significant SNP in our study) was associated with lean meat yield (LMY) and that two other SNPs close to the region between 36.15 and 38.56 Mb on OAR 6 were associated with carcass fat depth (FAT), intra-muscular fat (IMF) and dressing percentage (DRESS, calculated as the ratio of hot carcass weight to pre-slaughter weight) traits. In cattle, Setoguchi et al. [12] detected a QTL for carcass weight in a 591 kb interval on BTA6 and identified a causative gene variant in the *NCAPG* gene (NCAPG c.1326T > G, which changes the amino acid Ile442 to Met442 in the encoded protein). The *NCAPG* c.1326T > G variant is present in various bovine breeds and has been associated with birth weight [39], withers height and BW at adolescence [36, 40]. In Japanese Black cattle, Nishimura et al. [11] also identified a major QTL for carcass weight on BTA6. In horse, the *NCAPG-LCORL* locus is located on ECA3 (ECA for *Equus caballu*s chromosome) and a QTL located just upstream of the *LCORL* gene was found to be associated with height at the withers [5]. In our analysis, we also explored a larger region that surrounded the detected QTL and identified several relevant genes downstream of the QTL region i.e. *Kv channel-interacting protein 4* (*KCNIP4*), *G protein-coupled receptor 125* (*GPR125*) and *glucosidase, beta, acid 3* (*GBA3*). Recently, a region containing the chicken orthologous *KCNIP4* and *GPR125* genes was identified in the region between 71.6 and 80.2 Mb on GGA4 (GGA for *Gallus gallus* chromosome) and was shown to be associated with BW and average daily weight gain from 6 to 12 weeks [2].

Alignment of the *NCPAG-LCORL* region on OAR6 and syntenic regions in other mammalian species showed a high level of sequence similarity (Fig. 5), which supports both structural and functional conservation. This suggests that the orthologous genes that are located in a conserved region were maintained by evolution even after speciation from a common ancestor of the mammalian clade. Since there are multiple body size traits linked to the broader 2.41 Mb region in many mammalian species, it is likely that there is a common underlying biological mechanism, i.e. loss or gain of function of a single gene through species-specific mutations. Interestingly, this region is also associated with calving ease, which may represent an indirect trait for body size [41, 42] and fetal growth [39] in cattle and also with human birth weight [43]. These results suggest the action of a gene that is involved in the developmental processes before birth, and which continues to have an effect on the adult.

Several candidate genes are present in the 2.41 Mb region on OAR6, although some are unlikely due to the lack of direct syntenic relationships with the human genome. It is likely that body size traits associated with this region are associated with genes that are expressed in affected tissues such as skeletal muscle and to a lesser extent adipose tissue, both of which have interactive functions. Gene expression data for adipose tissue depots and skeletal muscle from ovine late gestation fetuses and lambs indicate that *LAP3*, *mediator complex subunit 28* (*MED28*), *family with sequence similarity 184, member B* (*FAM184B*), *DDB1 and CUL4 associated factor 16* (*DCAF16*), *NCAPG* and *LCORL* genes are all expressed in these tissues and associated with permissive chromatin marks (Vuocolo and Tellam, personal communication). Moreover, it has been demonstrated that the expression of *NCAPG* and *LCORL* in bovine adipose and muscle tissues is associated with feed intake and average daily gain [33]. By combining these data and the information described above, one can narrow down the list of positional candidate genes to *NCAPG* and *LCORL*.

*NCAPG* is involved in the condensation and stabilization of chromosomes during meiosis and mitosis. A non-synonymous mutation I442M (c.1326T > G) in the *NCAPG* gene is strongly associated with fetal growth and carcass traits in cattle [39], although this variant was excluded as a contributor to carcass weight in another cattle population [37]. Two studies have indirectly linked this *NCAPG* variant with arginine metabolism [40, 44]. Arginine has a major role in fetal and adult growth and more specifically in skeletal muscle growth [45, 46]. Close to the *NCAPG* gene is *LCORL*, which encodes a ligand-dependent nuclear receptor corepressor-like protein. Interestingly, in most mammals the amino-terminal region of this protein contains a poly-alanine tract, part of which is encoded by a pure trinucleotide repeat. More generally, this type of repeats is known to be susceptible to polymorphic variation [47], which often leads to altered protein function and disease. It is also often subject to natural selection, which may contribute to species-specific morphological differences [48–50]. Indeed, human expressed sequence tags provide evidence for polymorphic repeat expansions and contractions in this region of *LCORL*. The first exon of the ovine *LCORL* has not yet been annotated and it should be investigated for polymorphisms. Sequencing is currently in progress to identify the causal mutation.

## Conclusions

In summary, our GWAS identified 39 SNPs associated with body weight in sheep and we found a major QTL region that spans a 2.41 Mb region on OAR6. Within this region, the genes *NCAPG* and *LCORL* are likely candidate genes for BW. The region that harbors the *NCPAG* and *LCORL* genes on OAR6 is highly conserved among mammalian species and multiple body size traits in various species have been associated with this syntenic region, which may reflect that the underlying biological mechanisms share a common ancestry. These findings should facilitate the discovery of causal variants for BW and contribute to marker-assisted selection.

## Additional files

Additional file 1: Table S1. Number of SNPs before and after quality control and average distances between adjacent SNPs on each chromosome. (DOCX 16 kb) Additional file 2: Table S2. The 39 SNPs that have a significant association with body weight in 1743 Merino sheep. This table presents the chromosomal positions of 39 genome-wide significant SNPs with MAF, *P*-values as well as the proportion of genetic variance and allele substitution effects that can be attributed to each SNP. (DOCX 23 kb) Additional file 3: Table S3. Percentages of genetic variance explained by each chromosome. This table presents the percentages of genetic variance explained by each chromosome obtained by fitting all chromosomes simultaneously in the GCTA software. (DOCX 19 kb)
