# Supplementary material for: Genome-wide association study of body weight in Australian Merino sheep reveals an orthologous region on OAR6 to human and bovine genomic regions affecting height and weight
Source: Genet Sel Evol. 2015 Aug 14;47(1):66. doi: 10.1186/s12711-015-0142-4 (PMC4536601; doi:10.1186/s12711-015-0142-4)
Supplement: Additional file 1: Table S1. — Number of SNPs before and after quality control and average distances between adjacent SNPs on each chromosome. (DOCX 16 kb) [file 12711_2015_142_MOESM1_ESM.docx]

**Table S1. Number of SNPs before and after quality control and the average distances between adjacent SNPs on each chromosome**

| **Chromosome** | **Length of chromosome (Mbp)** | **No. SNPs** | | **Average distance (Kb)** | |
| --- | --- | --- | --- | --- | --- |
|  |  | **Before QC** | **After QC** | **Before QC** | **After QC** |
| 1 | 275.61 | 5930 | 5494 | 46.48 | 54.48 |
| 2 | 248.99 | 5474 | 5111 | 45.46 | 51.15 |
| 3 | 224.28 | 5008 | 4647 | 44.77 | 52.19 |
| 4 | 119.26 | 2680 | 2508 | 44.51 | 50.36 |
| 5 | 107.9 | 2363 | 2199 | 45.57 | 52.41 |
| 6 | 117.03 | 2592 | 2413 | 45.13 | 53.35 |
| 7 | 100.08 | 2252 | 2094 | 44.45 | 47.55 |
| 8 | 90.7 | 2057 | 1916 | 44.08 | 47.22 |
| 9 | 94.73 | 2141 | 1983 | 44.25 | 50.2 |
| 10 | 86.45 | 1851 | 1719 | 46.71 | 52.91 |
| 11 | 62.25 | 1180 | 1104 | 52.62 | 59.13 |
| 12 | 79.1 | 1723 | 1583 | 45.9 | 54.07 |
| 13 | 83.08 | 1696 | 1565 | 49 | 54.16 |
| 14 | 62.72 | 1174 | 1094 | 53.44 | 61.13 |
| 15 | 80.92 | 1694 | 1555 | 47.63 | 57.05 |
| 16 | 71.72 | 1580 | 1450 | 45.4 | 53.05 |
| 17 | 72.29 | 1420 | 1320 | 50.83 | 59.57 |
| 18 | 68.6 | 1413 | 1318 | 48.52 | 54.22 |
| 19 | 60.46 | 1248 | 1153 | 48.42 | 56 |
| 20 | 51.18 | 1148 | 1050 | 44.41 | 48.36 |
| 21 | 50.07 | 898 | 825 | 55.77 | 66.15 |
| 22 | 50.83 | 1097 | 1005 | 46.37 | 55.01 |
| 23 | 62.33 | 1128 | 1056 | 55.28 | 58.76 |
| 24 | 42.03 | 741 | 679 | 56.79 | 65.47 |
| 25 | 45.37 | 1001 | 931 | 45.34 | 48.66 |
| 26 | 44.08 | 924 | 868 | 47.68 | 50.56 |
