# Supplementary material for: Genome-wide association study of body weight in Australian Merino sheep reveals an orthologous region on OAR6 to human and bovine genomic regions affecting height and weight
Source: Genet Sel Evol. 2015 Aug 14;47(1):66. doi: 10.1186/s12711-015-0142-4 (PMC4536601; doi:10.1186/s12711-015-0142-4)
Supplement: Additional file 2: Table S2. — The 39 SNPs that have a significant association with body weight in 1743 Merino sheep. This table presents the chromosomal positions of 39 genome-wide significant SNPs with MAF, P-values as well as the proportion of genetic variance and allele substitution effects that can be attributed to each SNP. (DOCX 23 kb) [file 12711_2015_142_MOESM2_ESM.docx]

**Table S2. All 39 SNPs showing significant association with body weight in 1,743 Australian Merino sheep**

| SNP | Chr | Position | p-value | Adjusted  p-value | n0 | n1 | n2 | p | q | v_g_ | β (kg) |
| --- | --- | --- | --- | --- | --- | --- | --- | --- | --- | --- | --- |
| OAR6_41936490.1 | 6 | 37694563 | 2.37E-16 | 1.15E-11 | 119 | 706 | 918 | 0.27 | 0.73 | 6.17 | 2.12 |
| s17946.1 | 6 | 37164383 | 7.97E-14 | 3.87E-09 | 83 | 630 | 1030 | 0.23 | 0.77 | 5.88 | -2.13 |
| OAR6_41877997.1 | 6 | 37640732 | 2.48E-12 | 1.21E-07 | 110 | 650 | 983 | 0.25 | 0.75 | 6.05 | -1.88 |
| OAR3_128968872.1 | 3 | 120960429 | 2.16E-11 | 1.05E-06 | 313 | 861 | 569 | 0.43 | 0.57 | 5.86 | -1.63 |
| OAR6_41003295.1 | 6 | 36819342 | 2.40E-11 | 1.17E-06 | 150 | 722 | 871 | 0.29 | 0.71 | 6.50 | -1.74 |
| OAR14_57922732.1 | 14 | 57922732 | 3.21E-10 | 1.56E-05 | 22 | 321 | 1400 | 0.10 | 0.90 | 6.54 | 2.28 |
| OAR6_42945420.1 | 6 | 38567455 | 3.21E-10 | 1.56E-05 | 22 | 321 | 1400 | 0.10 | 0.90 | 6.54 | 2.28 |
| OAR12_19764133.1 | 12 | 16922406 | 5.82E-10 | 2.83E-05 | 288 | 853 | 602 | 0.41 | 0.59 | 6.49 | -1.48 |
| s41303.1 | 7 | 77974470 | 8.80E-10 | 4.28E-05 | 233 | 766 | 744 | 0.35 | 0.65 | 6.45 | -1.47 |
| OAR6_40449774.1 | 6 | 36234302 | 7.94E-09 | 3.86E-04 | 20 | 378 | 1345 | 0.12 | 0.88 | 6.62 | -2.08 |
| OAR14_33804119.1 | 14 | 32425979 | 1.10E-08 | 5.35E-04 | 36 | 433 | 1274 | 0.14 | 0.86 | 6.58 | -1.94 |
| OAR6_41558126.1 | 6 | 37334387 | 1.12E-08 | 5.44E-04 | 79 | 574 | 1090 | 0.21 | 0.79 | 6.30 | 1.69 |
| OAR10_28234413.1 | 10 | 28220524 | 1.23E-08 | 5.98E-04 | 362 | 839 | 542 | 0.45 | 0.55 | 6.94 | 1.35 |
| OAR3_170046187.1 | 3 | 158959518 | 2.14E-08 | 1.04E-03 | 118 | 655 | 970 | 0.26 | 0.74 | 6.52 | 1.54 |
| s10875.1 | 19 | 55289603 | 2.29E-08 | 1.11E-03 | 51 | 476 | 1216 | 0.17 | 0.83 | 6.31 | -1.69 |
| OAR3_79625099.1 | 3 | 75408156 | 2.54E-08 | 1.23E-03 | 125 | 634 | 984 | 0.25 | 0.75 | 5.60 | 1.47 |
| OAR6_40409402.1 | 6 | 36192023 | 3.30E-08 | 1.60E-03 | 9 | 254 | 1480 | 0.08 | 0.92 | 6.65 | 2.34 |
| OAR2_200781582.1 | 2 | 189390300 | 3.31E-08 | 1.61E-03 | 49 | 367 | 1327 | 0.13 | 0.87 | 6.35 | 1.85 |
| OAR6_40370293.1 | 6 | 36155169 | 3.39E-08 | 1.65E-03 | 110 | 641 | 992 | 0.25 | 0.75 | 6.25 | -1.59 |
| s64523.1 | 1 | 12775585 | 3.73E-08 | 1.81E-03 | 121 | 622 | 1000 | 0.25 | 0.75 | 6.21 | -1.48 |
| OAR6_42247197.1 | 6 | 37987281 | 4.04E-08 | 1.96E-03 | 60 | 512 | 1171 | 0.18 | 0.82 | 6.52 | -1.65 |
| OAR10_62872792.1 | 10 | 61341529 | 4.49E-08 | 2.18E-03 | 408 | 858 | 477 | 0.48 | 0.52 | 6.35 | 1.28 |
| s66267.1 | 23 | 58323699 | 4.72E-08 | 2.29E-03 | 237 | 781 | 725 | 0.36 | 0.64 | 6.46 | -1.30 |
| OAR12_2311264.1 | 12 | 3371387 | 5.44E-08 | 2.64E-03 | 44 | 510 | 1189 | 0.17 | 0.83 | 6.75 | -1.73 |
| OAR8_49852851.1 | 8 | 46382226 | 5.48E-08 | 2.66E-03 | 354 | 832 | 557 | 0.44 | 0.56 | 6.38 | 1.23 |
| OAR2_33892911.1 | 2 | 32570329 | 5.55E-08 | 2.70E-03 | 104 | 658 | 981 | 0.25 | 0.75 | 5.73 | -1.47 |
| OAR7_88735490.1 | 7 | 81383811 | 6.82E-08 | 3.31E-03 | 115 | 704 | 924 | 0.27 | 0.73 | 6.66 | 1.44 |
| OAR6_40724811_X.1 | 6 | 36522166 | 7.41E-08 | 3.60E-03 | 18 | 385 | 1340 | 0.12 | 0.88 | 6.26 | -1.91 |
| OAR1_141580492.1 | 1 | 130797358 | 1.07E-07 | 5.20E-03 | 267 | 829 | 647 | 0.39 | 0.61 | 6.39 | -1.27 |
| s36305.1 | 13 | 61597135 | 1.08E-07 | 5.25E-03 | 34 | 415 | 1294 | 0.14 | 0.86 | 7.05 | -1.79 |
| OAR7_81534131.1 | 7 | 74570199 | 1.11E-07 | 5.39E-03 | 313 | 855 | 575 | 0.42 | 0.58 | 6.44 | 1.27 |
| s29813.1 | 14 | 52182942 | 1.11E-07 | 5.39E-03 | 413 | 898 | 432 | 0.49 | 0.51 | 7.10 | 1.29 |
| s43545.1 | 15 | 74500105 | 1.15E-07 | 5.59E-03 | 1 | 92 | 1650 | 0.03 | 0.97 | 6.57 | -3.49 |
| OAR6_40855809.1 | 6 | 36655091 | 1.16E-07 | 5.64E-03 | 53 | 448 | 1242 | 0.16 | 0.84 | 6.34 | 1.61 |
| s40612.1 | 2 | 182039142 | 1.24E-07 | 6.03E-03 | 281 | 823 | 639 | 0.40 | 0.60 | 6.34 | 1.26 |
| s35115.1 | 3 | 14572165 | 1.42E-07 | 6.90E-03 | 61 | 454 | 1228 | 0.17 | 0.83 | 6.01 | 1.70 |
| s38098.1 | 14 | 23489233 | 1.70E-07 | 8.26E-03 | 18 | 327 | 1398 | 0.10 | 0.90 | 6.24 | -2.02 |
| OAR6_41768532.1 | 6 | 37533664 | 1.85E-07 | 8.99E-03 | 64 | 521 | 1158 | 0.19 | 0.81 | 6.46 | 1.54 |
| s05777.1 | 8 | 30348320 | 2.00E-07 | 9.72E-03 | 89 | 626 | 1028 | 0.23 | 0.77 | 6.34 | 1.48 |

A total of 39 SNPs were identified as genome-wide significant for a Bonferroni-corrected p-value threshold of 1.15x10^-11^. *Vg* indicates the proportion of the genetic variance attributable to each SNP estimated by ASReml. *β* is the allele substitution effect. SNP positions are based on Oar_v3.1 assembly of the ovine genome sequence.
