# Supplementary material for: Genome-wide association study of body weight in Australian Merino sheep reveals an orthologous region on OAR6 to human and bovine genomic regions affecting height and weight
Source: Genet Sel Evol. 2015 Aug 14;47(1):66. doi: 10.1186/s12711-015-0142-4 (PMC4536601; doi:10.1186/s12711-015-0142-4)
Supplement: Additional file 3: Table S3. — Percentages of genetic variance explained by each chromosome. This table presents the percentages of genetic variance explained by each chromosome obtained by fitting all chromosomes simultaneously in the GCTA software. (DOCX 19 kb) [file 12711_2015_142_MOESM3_ESM.docx]

**Table S3. The percentage of genetic variance explained by each chromosome**

| OAR | Length (Mb) | Explained variance | SE | LRT |
| --- | --- | --- | --- | --- |
| 1 | 275.61 | 1.85 | 0.03 | 196.55 |
| 2 | 248.99 | 1.42 | 0.03 | 174.27 |
| 3 | 224.28 | 2.64 | 0.03 | 201.89 |
| 4 | 119.26 | 1.78 | 0.02 | 170.21 |
| 5 | 107.9 | 1.72 | 0.02 | 137.00 |
| 6 | 117.03 | 7.71 | 0.03 | 197.89 |
| 7 | 100.08 | 0.96 | 0.02 | 146.22 |
| 8 | 90.7 | 4.47 | 0.02 | 146.94 |
| 9 | 94.73 | 0.84 | 0.02 | 150.94 |
| 10 | 86.45 | 3.35 | 0.02 | 161.66 |
| 11 | 62.25 | 8.54 | 0.02 | 171.35 |
| 12 | 79.1 | 8.91 | 0.03 | 179.09 |
| 13 | 83.08 | 1.46 | 0.02 | 128.49 |
| 14 | 62.72 | 2.73 | 0.02 | 142.41 |
| 15 | 80.92 | 0.00 | 0.02 | 117.15 |
| 16 | 71.72 | 2.02 | 0.02 | 134.54 |
| 17 | 72.29 | 0.00 | 0.02 | 101.71 |
| 18 | 68.6 | 0.00 | 0.02 | 117.04 |
| 19 | 60.46 | 0.58 | 0.02 | 105.54 |
| 20 | 51.18 | 2.12 | 0.02 | 114.54 |
| 21 | 50.07 | 0.00 | 0.02 | 103.32 |
| 22 | 50.83 | 0.00 | 0.02 | 106.36 |
| 23 | 62.33 | 3.71 | 0.02 | 131.95 |
| 24 | 42.03 | 2.92 | 0.02 | 95.49 |
| 25 | 45.37 | 5.74 | 0.02 | 148.08 |
| 26 | 44.08 | 1.50 | 0.02 | 95.45 |

Explained variance is given in percentage and was standardized according to each chromosome’s length. SE is the standard error. Likelihood ratio test (LRT) was calculated in GCTA fitting each chromosome individually.
